# A Dye-Assisted Paper-Based Assay to Rapidly Differentiate the Stress of Chlorophenols and Heavy Metals on *Enterococcus faecalis* and *Escherichia coli*

**DOI:** 10.3390/bios13050523

**Published:** 2023-05-06

**Authors:** Wanqing Dai, Bibi Inumbra, Po Yu Wong, Alma Sarmiento, Ying Yau, Jie Han, Guozhu Mao, Yung-Kang Peng, Jian Lin Chen

**Affiliations:** 1 Department of Applied Science, School of Science and Technology, Hong Kong Metropolitan University, Good Shepherd Street, Ho Man Tin, Hong Kong SAR, China; 2 School of Environmental Science and Engineering, Tianjin University, Tianjin 300350, China; 3 Department of Chemistry, City University of Hong Kong, Tat Chee Avenue, Kowloon, Hong Kong SAR, China; 4 State Key Laboratory of Marine Pollution, City University of Hong Kong, Tat Chee Avenue, Kowloon, Hong Kong SAR, China

**Keywords:** paper-based PAD, toxicity, chlorophenol, heavy metal

## Abstract

Biological toxicity testing plays an essential role in identifying the possible negative effects induced by substances such as organic pollutants or heavy metals. As an alternative to conventional methods of toxicity detection, paper-based analytical device (PAD) offers advantages in terms of convenience, quick results, environmental friendliness, and cost-effectiveness. However, detecting the toxicity of both organic pollutants and heavy metals is challenging for a PAD. Here, we show the evaluation of biotoxicity testing for chlorophenols (pentachlorophenol, 2,4-dichlorophenol, and 4-chlorophenol) and heavy metals (Cu^2+^, Zn^2+^, and Pb^2+^) by a resazurin-integrated PAD. The results were achieved by observing the colourimetric response of bacteria (*Enterococcus faecalis* and *Escherichia coli*) to resazurin reduction on the PAD. The toxicity responses of *E. faecalis*-PAD and *E. coli*-PAD to chlorophenols and heavy metals can be read within 10 min and 40 min, respectively. Compared to the traditional growth inhibition experiments for toxicity measuring which takes at least 3 h, the resazurin-integrated PAD can recognize toxicity differences between studied chlorophenols and between studied heavy metals within 40 min.

## 1. Introduction

Persistent organic pollutants (POPs) are chemicals of global concern due to their unique combination of physical and chemical properties as well as their significant negative effects on human health and the environment [1]. They exist in a stable state with high lipid solubility [2,3], leading to their accumulation and the subsequent accumulation of toxicity [2,4]. Heavy metal in the environment is also of concern due to its non-degradability and accumulation in water [5]. Exposure to both POPs and heavy metals is considered serious a health risk [6]. Hence, detecting the toxicity of such substances in the environment quickly and accurately is critical to assess their risk.

There are various conventional analytical techniques for the detection of organic and heavy metal pollutants, such as High-performance liquid chromatography (HPLC), Gas chromatography (GC), atomic absorption spectroscopy (AAS), mass spectrometry (MS), etc. These analytical techniques are limited by the investment costs, the necessity of skilled labor during operation, sample pre-processing, and lack of real-time capability [7]. Paper is a versatile and cost-effective medium that is easy to transport. The advent of microfluidic paper-based analytical devices (PADs) can simultaneously satisfy on-site detection while requiring fewer samples, providing ease of operation and requiring no external power, making these devices increasingly useful in biomedicine [8], environmental testing [9,10], and food safety [11]. The integration of colourimetric detection with PAD is one of the most used detection methods due to its visibility, excellent stability, rapid detection capabilities, and ease of use in the field. Various colorimetric PAD assays have been reported, with the change in color of the PAD before and after the test. The Griess reagent was used to integrate into the PAD, which then combined with nitrite to produce an azo dye, visible as a magenta hue [10]. To detect heavy metal ions such as Ni (II), Cr (VI), and Cu (II) in water samples, color alterations before and after the indicator–metal complex system were utilized [12,13]. The utilization of silver nanoparticles in conjunction with PAD devices has been demonstrated as a viable method for detecting cyanide (CN) in water samples. This was achieved by monitoring the color alteration of silver nanoparticles-PAD from yellow to colorless [14]. In addition, gold nanoparticles were also reported to be used in the colorimetric PADs in conjunction with digital images to measure color responserepresenting additional advantage [15].

Resazurin is commonly used as an oxidation−reduction indicator, formulated into in vitro toxicology assay kits (i.e., AlamarBlue). The reduction in blue resazurin to pink resorufin, then further to colorless dihydroresorufin (resazurin→resorufin⇄dihydroresorufin) is the response to intercellular metabolic activity [16]. Therefore, it is widely used as an indicator of microbial growth and antibacterial properties [17,18], antibiotic susceptibility testing [16], and cytotoxicity [19,20,21]. A microfluidic droplet reactor with resazurin reduction assay has been utilized to detect heavy metals [22,23] and hydrophobic toxicants [24] that inhibit *E. faecalis* metabolism. However, the microfluidics system is not portable and requires exterior force to drive the liquid. As a solution to these limitations, in this present study, resazurin reduction assay was integrated with PAD devices to differentiate the biotoxicity of chlorophenols (pentachlorophenol (PCP), 2,4-dichlorophenol (2,4-DCP), 4-chlorophenol (4-CP)) and heavy metals (Pb^2+^, Zn^2+^, and Cu^2+^) on *E. coli* and *E. faecalis*. The kinetics of color change in resazurin on PAD captured by a smartphone was used to measure the bio-toxicity of studied substances. By comparing these results to the traditional biotoxicity measures, we proposed that PAD integrating resazurin reduction assay is a novel and efficient method to study the stress of toxicants on microorganisms.

## 2. Materials and Methods

### 2.1. Chemicals and Materials

Brain heart infusion (BHI) broth was obtained from Hopebio, Qingdao, China. Several of the chemicals used in this work were purchased from Sigma-Aldrich, HK, including PCP, 2,4-DCP, 4-CP, and Lead (II) nitrate (≥99%). Zinc acetate and copper (II) sulphate pentahydrate were purchased from VWR Chemicals, Radnor, PA, USA. Resazurin sodium salt (90%) was obtained from J&K Scientific, Beijing, China. Whatman chromatography paper (WCP) was selected from Whatman, Hong Kong.

### 2.2. Fabrication of PADs

WCP used in this experiment was sliced into strips at the size of 3 × 50 mm. The strips were soaked in 0.2 mM resazurin solution for 10 min and then placed in a glass petri dish with a lid and allowed to air dry overnight in a fume hood under aphotic and ambient conditions. The dry WCP strips were stored in a desiccator for later use as resazurin-integrated PADs.

### 2.3. Bacterial Culture

*E. faecalis* (ATCC 6569) and *E. coli* (ATCC 25922) were stored at −80 °C. The volume of 600 μL of the bacterial stock solution was placed in 10 mL of BHI for subculture and grown at 37 °C and 150 rpm until the optical density (OD_600nm_) was 1.4, (3.5 h for *E. faecalis*, ~9 × 10^8^ CFU/mL; 6 h for *E. coli*, ~2 × 10^9^ CFU/mL). For subsequent experiments, the culture medium was diluted with BHI broth to OD_600nm_ = 0.85 (for *E. faecalis*, ~1 × 10^8^ CFU/mL; for *E. coli*, ~9 × 10^8^ CFU/mL) and 0.45 (for *E. faecalis*, ~8 × 10^7^ CFU/mL; for *E. coli*, ~4 × 10^8^ CFU/mL) and measured with a UV–vis spectrophotometer.

### 2.4. PAD Toxicity Detection Method and Data Analysis

Three types of chlorophenols, PCP, 2,4-DCP, and 4-CP, were spiked in the bacterial culture prepared in Section 2.3 to yield final concentrations of 10 mg/L, respectively. Thirty μL chlorophenol-spiked bacterial culture at OD_600nm_ of 1.4, 0.85, and 0.45 was, respectively, dropped onto PADs prepared in Section 2.2. A smartphone was used to record the color changes on the PAD. Meanwhile, a positive control group with bacterial culture without toxicants addition was set up and a blank control only with BHI was also set up. Every 10 min, photos were snapped in the recorded video and then imported into the application *Color Picker* to quantify the hue value. The increment of the hue value (ΔHue) was obtained by subtracting the initial hue value (time = 0 min).

The kinetic reduction equation for resazurin was the basis for this experiment. It is used as a toxicity indicator when comparing the toxicity of toxic substances with *E. coli*. The reduction in resazurin by living cells: $Resazurin\left(blue\right)+M1\overset{k_{1}}{\to }Resorufin\left(pink\right)+M2\overset{k_{2}}{\Leftrightarrow }dihydroresorufin\left(transparent\right)$ (M1 and M2 here represent reducing substances such as NADH/NADPH in the living cells). The colorimetric based on resazurin used as a toxicity indicator when comparing the toxicity of toxic substances with *E. coli* and can be described by Equation (1) [25,26]:

(1) $$∆Hue=∆{Hue}_{0}\frac{k_{1}}{k_{2}-k_{1}}\left(e^{-k_{1}\left[M\right]t}-e^{-k_{2}\left[M\right]t}\right).$$

The value of *k*_1_ and *k*_2_ can be obtained by fitting chromaticity values to the reaction reduction Equation (1).

### 2.5. Applicability for Heavy Metal Toxicity Detection

To verify the feasibility of this PAD technique in the detection of heavy metal toxicity, copper, zinc, and lead were chosen as proxies, whose concentrations were determined according to IC_50_ values published in literatures [27,28,29]. The copper (II) sulphate pentahydrate, zinc (II) acetate, and lead (II) nitrate solutions were spiked in the bacterial culture with final metal ion concentrations of 7.00, 10.00, and 18.00 mg/L, respectively. Thirty μL metal-spiked bacterial culture was dropped onto the resazurin-integrated PAD prepared in Section 2.3. Two groups of control were set up and the same experiment was carried out in the same way as that mentioned in Section 2.4.

### 2.6. Traditional Toxicity Detection

To evaluate the biotoxicity test PAD, a traditional growth curve method was conducted in BHI broth to confirm the toxicity of chlorophenols, and heavy metals used above at the relevant concentrations, in which another 0.4 mL of subcultured bacteria (*E. faecalis* and *E. coli*, respectively) was mixed with sterile BHI broth in 100 mL serum bottles. Chlorophenols and heavy metals were then spiked, respectively, to result in the concentrations used above. Positive controls were also conducted. Serum vials were sealed, and they were incubated at 37 °C. OD_600nm_ was monitored until it reached the stationary phase at regular intervals.

## 3. Results and Discussion

Both *E. faecalis* (Gram-positive) and *E. coli* (Gram-negative) were common facultative anaerobic bacteria inhabiting the human gastrointestinal tract, and their isolation and culture were frequently used as indicators of bacterial toxicity [30]. The videos of resazurin reduced by *E. faecalis* and *E. coli* on the PAD were sped up by a factor of 600 (details can be found in Videos S1–S4). As the bacterial culture solution was dripped onto the paper strip, the color changed from blue to pink, meaning the bacteria reduced resazurin to resorufin. Furthermore, the color changed at different rates under certain conditions, such as different species of bacteria, bacterial densities, etc.

### 3.1. The Kinetics of Color Change on PADs

**Figure 1:**
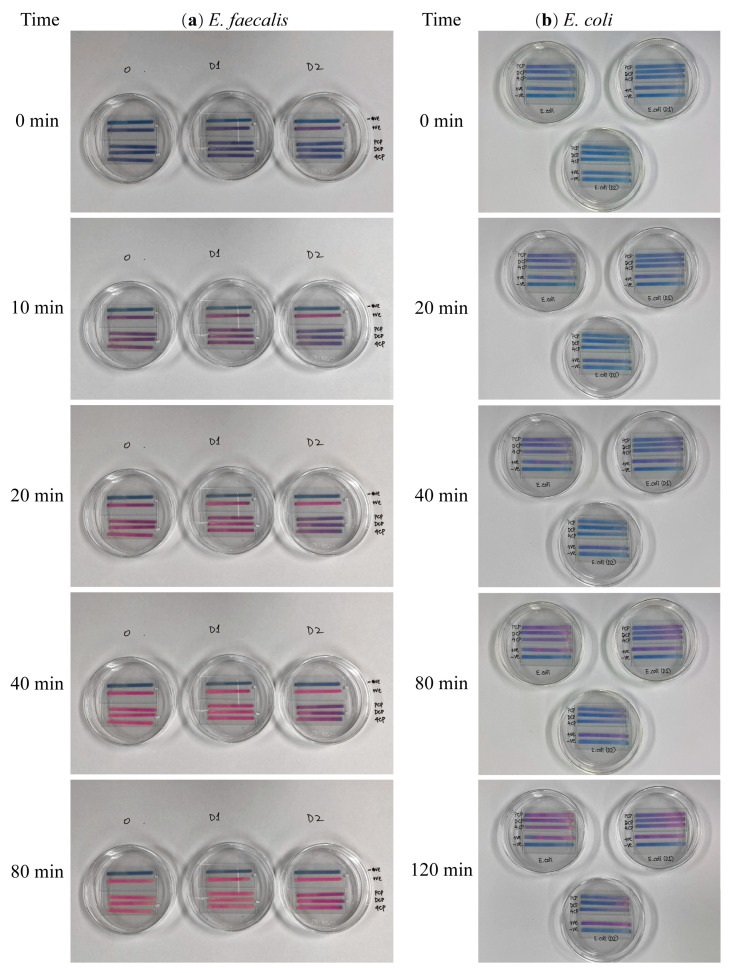
Resazurin reaction with PADs of *E. faecalis* (**a**) and *E. coli* (**b**) under chlorophenols stress. The block control (BHI), positive control (BHI + bacteria), PCP, 2,4-DCP, and 4-CP experimental groups were listed from top to bottom of each dish (For *E. faecalis*, 0 represents undiluted bacterial density, D1 represents bacterial density diluted two times, OD_600nm_ = 0.85, and D2 represents bacterial density diluted four times, OD_600nm_ = 0.45. For the *E. coli* on the right, the D1 and D2 marks represent that *E. coli* was diluted 2 and 4 times, respectively, and the OD_600nm_ corresponds to 0.85 and 0.45. In addition, the petri dish labeled with *E. coli* represents undiluted bacteria).

**Figure 2:**
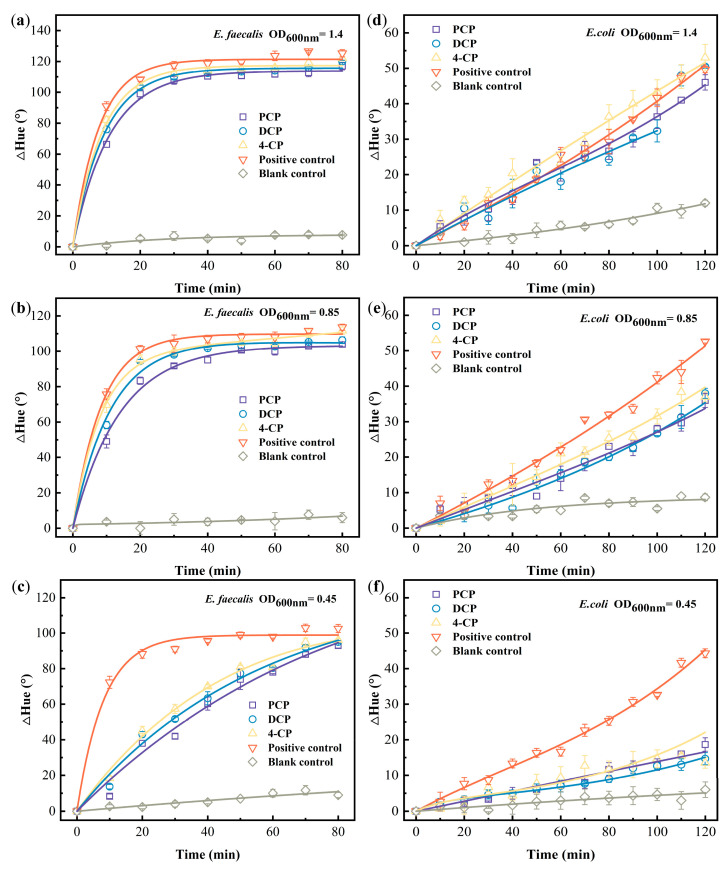
Color kinetic changes in three chlorophenols on the growth of *E. faecalis* and *E. coli*. The reduction equation for resazurin fits *E. faecalis* and *E. coli* exposed to chlorophenols compounds. (**a**) *E. faecalis* with OD_600nm_ = 1.4, (**b**) *E. faecalis* with OD_600nm_ = 0.85, and (**c**) reduction process of resazurin by *E. faecalis* with OD_600nm_ = 0.45 under chlorophenols stress (**d**) *E. coli* with OD_600nm_ = 1.4 (the last two outliers were not involved in the fitting), (**e**) *E. coli* with OD_600nm_ = 0.85, and (**f**) *E. coli* with OD_600nm_ = 0.45 during the reduction in resazurin under chlorophenols stress.

The pictures captured in the recorded video to illustrate the color change in reducing resazurin by *E. faecalis* and *E. coli* (Figure 1). The difference in hue values every ten 10 from those at 0 min were fitted in conjunction with Equation (1) (Figure 2). Table 1 summarizes the simulation results (*k*_1_ and *k*_2_). The majority of *E. faecalis* fitting results were more than 0.9 (>0.636, critical *R*^2^ under *p* = 0.01). In general, the fitting outcomes for *E. coli* were higher than 0.9, which is higher than 0.467 (critical *R*^2^ under *p* = 0.01). These results suggest that the model (Equation (1)) can reasonably be used to simulate the reduction process of resazurin on PADs for two different strains of bacteria, *E. faecalis* and *E. coli*.

### 3.2. For Bacterial Species

Two strains of bacteria under different densities were tested for their ability to reduce resazurin on the PAD. It can be seen in Figure 2a–c that the color change in positive control (*E. faecalis* with BHI) reaches equilibrium at about 30 min, indicating that resorufin has reached its maximum concentration in the *E. faecalis* system. For *E. coli*, the ΔHue value of the positive control showed an increasing trend during the detection time (Figure 2d–f), indicating that resazurin was still being reduced in *E. coli* system. The reduction in resazurin requires attachment to bacteria, diffusion through cell walls and membranes, and intracellular metabolism [31]. It can also be seen in Table 1 that the *k*_1_ value of *E. faecalis* fitted with different bacterial densities was between 0.35 and 0.59, while that of *E. coli* was 0.0037–0.041, revealing faster kinetics of resazurin reduction by *E. faecalis* than by *E. coli*.

It is believed that Gram-positive bacteria have monolayer cell walls that are composed primarily of peptidoglycan and acidic polysaccharides, such as teichoic acid. Gram-negative bacteria contain multilayered cell walls and lipopolysaccharide (LPS)-containing outer membrane [32]. The presence of large numbers of LPS makes the cell membrane less permeable, resulting in slowing down the diffusion of dye into the cells [33]. It was reported that resazurin was reduced to resazurin within the bacteria [34]. By adsorbing resazurin to the bacteria, resazurin passes through the cell wall and membrane, then reacts with intracellular metabolites [26]. According to Equation (1), [M] represents the reducing substance, such as NADH and NADPH, in living bacteria. The content of resazurin (meaning of ∆Hue_0_) that enters the bacteria through the bacterial membrane is relatively fixed due to the limited absorption sites on the bacterial membrane [26]. Because of the different bacterial structures of *E. faecalis* and *E. coli*, the content of resazurin entering the bacteria will be different due to the difference in the speed of resazurin passing through the bacterial cell wall/membrane. The kinetics under the same bacterial density (positive control fitting curve, the red one, in Figure 2) and the simulated *k*_1_ value demonstrate that *E. faecalis* reduces resazurin faster than *E. coli.*

### 3.3. For Bacterial Density

**Figure 3:**
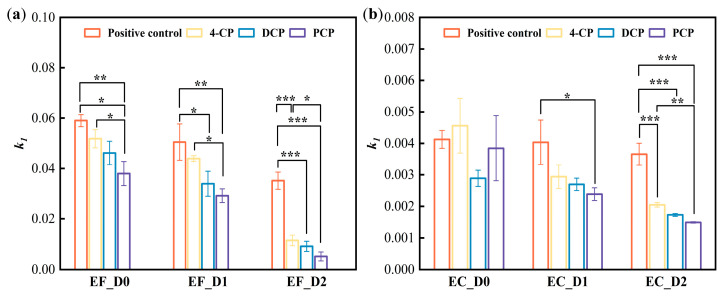
Rate constants of resazurin reduction by *E. faecalis* and *E. coli* under chloroform stress. (**a**) The *k*_1_ values of resazurin reduction on *E. faecalis*-PADs and their differences were analyzed, and (**b**) the *k*_1_ values of resazurin reduction on *E. coli*-PADs and their differences were analyzed. D0 represents undiluted bacterial density, D1 represents bacterial density diluted twice, OD_600nm_ = 0.85, and D2 represents a 4-fold dilution of bacterial density, OD_600nm_ = 0.45. In addition, the ANOVA’s turkey test was applied to variance analysis (* *p* < 0.05, ** *p* < 0.01, *** *p* < 0.001 compared in groups with the same dilution).

The simulated *k*_1_ values based on Figure 2 are summarized in Table 1. *E. faecalis* density at OD_600nm_ 1.4 and 0.85 had the *k*_1_ value of 0.059 and 0.050 min^−1^, respectively. Although these two densities were different, the amount of bacteria in the culture medium remained in the same order of magnitude (~10^8^), resulting in nonsignificant resazurin reduction kinetics difference between them. When the bacterial culture was further diluted (four times) to achieve density at OD_600nm_ 0.45, the resazurin reduction kinetics significantly slowed down (*k*_1_ at 0.035 min^−1^). The same phenomenon manifested in both the control and chlorophenol-treated groups (Figure 2a–c), whose statistical analysis results are shown in Figure 3a. Although the addition of chlorophenols impacted *E. faecalis* and its reducing resazurin on PAD at densities of 1.4 and 0.85, it was unable to identify chlorophenols’ toxicity difference. Meanwhile, when *E. faecalis* density was at 0.45, according to the fitted *k*_1_ value in Table 1 and statistical analysis in Figure 3a, the toxicity of the three chlorophenols could be sorted as PCP > 2,4-DCP > 4-CP, which was closely correlated with the number of substituted chlorine atoms [35] and the same as the toxicity sequence of various chlorophenols [36]. Hence, OD_600nm_ 0.45 was recommended as *E. faecalis* density for further study.

Compared with *E. faecalis*, the color changed slowly as different densities of *E. coli* were dripped onto PADs. According to the process of resazurin reduced by *E. coli* (Figure 2d–f), the corresponding fitted *k*_1_ value in Table 1 and statistical analysis in Figure 3b, *E. coli* density at 1.4 and 0.85 did not make resazurin reduction kinetics significant among PCP, 2,4-DCP, and 4-CP. When *E. coli* density was at 0.45, the same as that of *E. faecalis*, the toxicity of those three chlorophenols was differentiated. However, this process required at least 80 min, which is longer than that required for *E. faecalis* system (i.e., 30 min). Therefore, OD_600nm_ 0.45 was used as the bacterial density for subsequent *E. coli* detection of heavy metal toxicity. It showed sensitivity as two different types of bacteria were diluted four-fold to show a difference in toxicity instead of an order of magnitude dilution in traditional measurement.

### 3.4. Comparison with Traditional Toxicity Detection Methods

**Figure 4:**
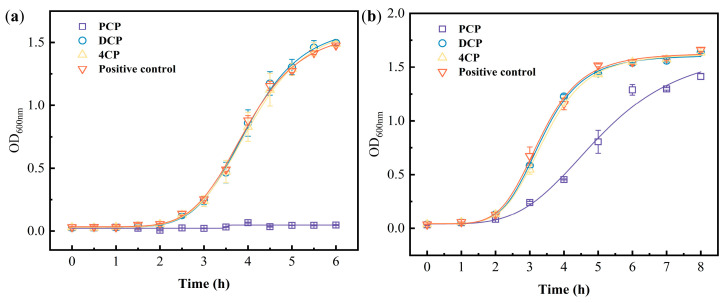
The growth curve of *E. faecalis* (**a**) and *E. coli* (**b**) under the stress of chlorophenols (conventional growth inhibition assay); the growth curves were obtained by logistic fitting.

Incubated with 10 mg/L PCP, the growth of Gram-positive *E. faecalis* was obviously affected (Figure 4a), indicating that the bacteria cannot grow. However, 10 mg/L of 2,4-DCP and 4-CP did not show an effect on the growth of *E. faecalis*. The traditional absorbance measurements cannot completely differentiate the toxicity of 10 mg/L PCP, 2,4-DCP, and 4-CP. Meanwhile, by the PADs developed in this present study, the inhibition of 10 mg/L PCP, 2,4-DCP, and 4-CP can be differentiated in 30 min. 

For Gram-negative *E. coli*, compared with the control group, the growth curve of 10 mg/L PCP showed a certain inhibitory effect (Figure 4b). Unlike what happened with *E. faecalis*, PCP partially inhibited the growth of *E. coli*, revealing *E. coli* resistance to potentially toxic substances. In contrast to the conventional method, which requires 4 h to demonstrate chlorophenol impact on *E. coli* growth, resazurin-integrated PADs can demonstrate chlorophenol inhibition effect on this Gram-positive strain in 80 min.

### 3.5. Toxicity Testing for Heavy Metals

**Figure 5:**
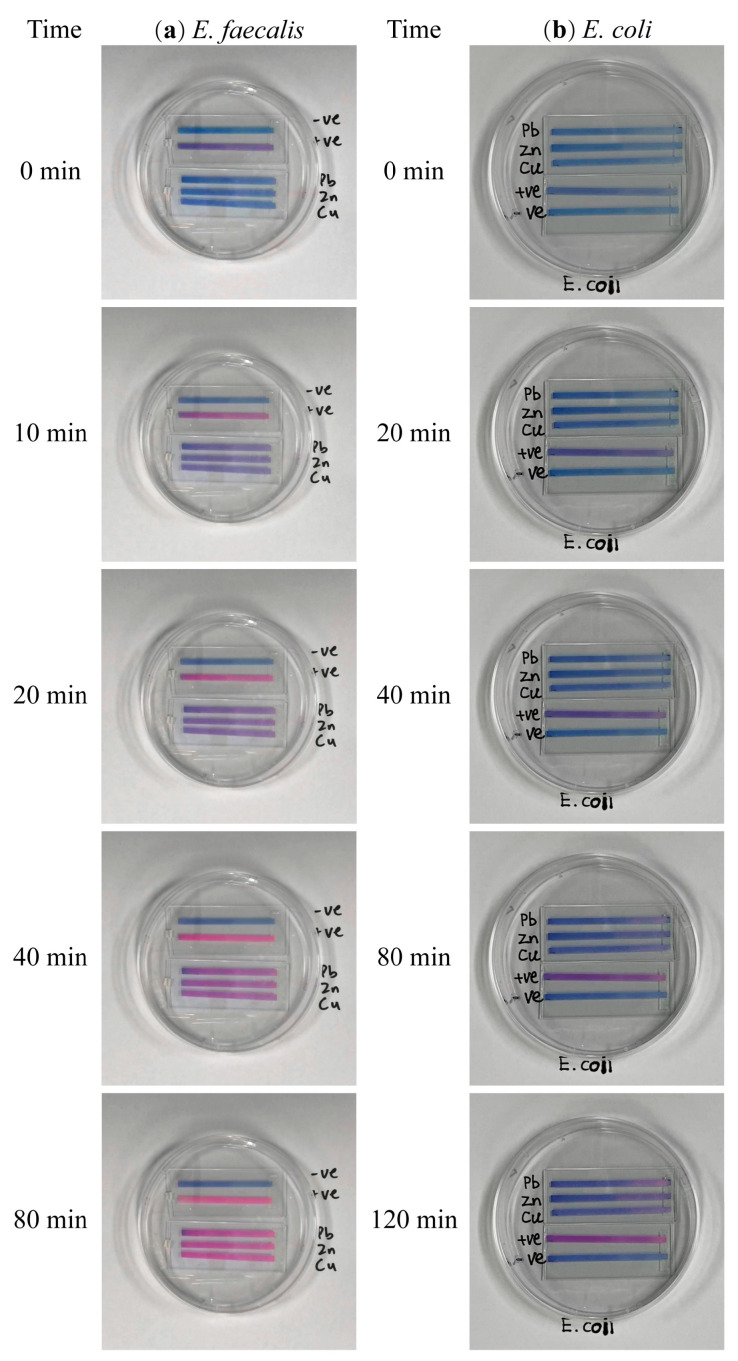
Color change in resazurin on PADs of *E. faecalis* (**a**) and *E. coli* (**b**) under heavy metal stress. (**a**) For *E. faecalis*-PAD, from top to bottom, are the experimental groups, the negative control (BHI), the positive control (BHI + bacteria), and the Pb, Zn, and Cu control groups. (**b**) For *E. coli*-PAD, from top to bottom, are Pb, Zn, and Cu experimental groups, positive control (BHI + bacteria), and blank control (BHI).

The toxic effects of heavy metals on both *E. faecalis* and *E. coli* were detected by the resazurin-integrated PADs developed above at an OD_600nm_ 0.45. According to the color change on PADs (Figure 5), the obtained data (ΔHue) were fitted to Equation (1). The simulation results (*k*_1_ and *k*_2_ values) were summarized in Table 2. The *R*^2^ of *E. faecalis* fitting results were more than 0.97 (>0.636, critical *R*^2^) and the *R*^2^ of *E. coli* fitting results were around 0.9 (>0.467, critical *R*^2^). This suggested that the model (Equation (1) can still well reflect the resazurin reduction process carried out by *E. faecalis* and *E. coli* on PADs in the presence of heavy metals.

**Figure 6:**
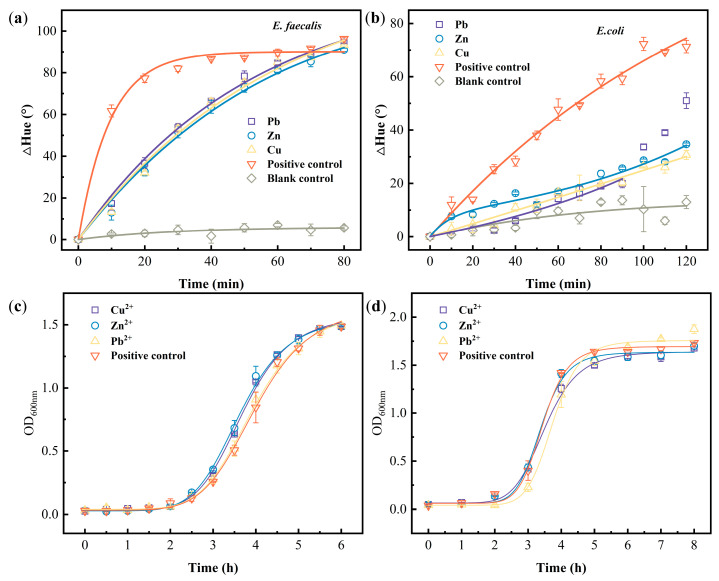
Color kinetic curve and growth curve of heavy metal ions on the growth of *E. faecalis* and *E. coli*. (**a**) Color kinetic change of the *E. faecalis*-PAD, (**b**) color kinetic change of the *E. coli* -PAD (data from 100–120 min for Pb in (**b**) were not included in the fitting process), (**c**) the conventional growth curve of *E. faecalis* under the stress of metal ions. (**d**) The traditional growth curve of *E. coli* under the stress of metal ions.

When using *E. faecalis* as a model, spiking three heavy metals (Cu^2+^, Zn^2+^, and Pb^2+^) made the color change slower than the positive control group (Figure 5a). The ΔHue values showed that the positive control group reached equilibrium at 30 min, while the heavy metal-spiked group did not reach it during the entire recording time (Figure 6a), showing heavy metal toxicity and suggesting that the presence of heavy metals inhibits bacterial activity and hence retards the resazurin reduction on PADs. Comparing the *k_1_* value in Table 2, there was no statistical difference in the reduction rate of resazurin on PADs among Cu^2+^, Zn^2+^, and Pb^2+^ because the concentrations of these three heavy metals were all at IC_50_ levels whose order was Cu^2+^ < Zn^2+^ < Pb^2+^. Hence, the toxicities of the three heavy metals were as reported [28]: Cu^2+^> Zn^2+^ > Pb^2+^. However, the traditional method did not detect the toxicity of the three heavy metals to *E. faecalis* (Figure 6c).

With the same concentrations, Cu^2+^, Zn^2+^, and Pb^2+^ were spiked into *E. coli* culture to evaluate the stress of heavy metals on such Gram-negative bacteria by growth-based inhibition method and resazurin-integrated PADs, respectively. The growth-based inhibition method cannot reveal the toxicity of those three heavy metals to *E. coli* (Figure 6d), similar to the finding for *E. faecalis*. However, the color change on resazurin-integrated PADs (Figure 5b and Figure 6b) suggests that Cu^2+^, Zn^2+^, and Pb^2+^ at IC_50_ level show the same toxicity to *E. coli*. Comparing the *k*_1_ value in Table 2, although there was no statistical difference among Cu^2+^, Zn^2+^, and Pb^2+^’s *k*_1_ values on *E. coli*, their toxicity can be checked in 40 min (Figure 6b).

## 4. Conclusions

The toxicity of several toxic substances to two types of bacteria, Gram-positive bacteria (*E. faecalis*) and Gram-negative bacteria (*E. coli*), was investigated by resazurin-integrated PADs. The color change on PADs and the kinetics of the resazurin reaction were used to assess the biotoxicity. Bacterial density is a key factor affecting the sensitivity of PADs’ toxicity detection. Results show that OD_600nm_ 0.45 of *E. faecalis* and *E. coli* can differentiate the stress of PCP, 2,4-DCP, and 4-CP within 10 and 40 min, respectively. To further verify the applicability of this resazurin-integrated PADs, heavy metals, Cu^2+^, Zn^2+^, and Pb^2+^ were used as toxicants for their biotoxicity detection. Similarly, according to the kinetics of resazurin reaction on PADs based on color change, the toxic effects of heavy metals on *E. faecalis* can be observed in only 10 min. All these results show that this resazurin-integrated PADs technology can detect the stress of toxic substances, including organic pollutants and heavy metals, in a gross way. The resazurin-based color response is targeted against reducing substances in living bacteria. The lack of selectivity is a limitation of resazurin-PAD. Integrating its sensitivity for toxicity differentiation, future work will involve modifying resazurin to make this resazurin-based assay selective for some important pollutants to study their synergistic or antagonistic or additive effects.

## Figures and Tables

**Table 1:** The *k*_1_ and *k*_2_ values of the resazurin reduction model fitted by *E. faecalis* and *E. coli* under different bacterial densities and chlorophenols.

Bacteria	Bacterial Density (OD_600nm_)	Group	*k*_1_ ^#^	*k*_2_ ^#^	*R*_2_ *
*E. faecalis* (n = 9)	1.4	Positive control	0.059 ± 0.002	1.04 × 10^−18^ ± 6.20 × 10^−19^	0.998
		PCP	0.038 ± 0.005	1.67 × 10^−18^ ± 1.80 × 10^−18^	0.994
		2,4-DCP	0.046 ± 0.005	6.62 × 10^−21^ ± 5.32 × 10^−18^	0.995
		4-CP	0.052 ± 0.004	2.41 × 10^−21^ ± 3.40 × 10^−21^	0.994
	0.85	Positive control	0.050 ± 0.007	6.18 × 10^−25^ ± 4.12 × 10^−25^	0.989
		PCP	0.029 ± 0.003	−1.41 × 10^−5^ ± 1.96 × 10^−4^	0.991
		2,4-DCP	0.034 ± 0.005	8.10 × 10^−5^ ± 1.05 × 10^−4^	0.987
		4-CP	0.044 ± 0.001	6.76 × 10^−23^ ± 3.15 × 10^−23^	0.991
	0.45	Positive control	0.035 ± 0.003	6.61 × 10^−22^ ± 9.07 × 10^−22^	0.982
		PCP	0.005 ± 0.001	5.21 × 10^−3^ ± 1.46 × 10^−3^	0.962
		2,4-DCP	0.009 ± 0.002	5.04 × 10^−3^ ± 3.91 × 10^−3^	0.981
		4-CP	0.013 ± 0.001	5.52 × 10^−3^ ± 6.10 × 10^−3^	0.984
*E. coli* (n = 13)	1.4	Positive control	0.0041 ± 0.000	−6.15 × 10^−3^ ± 1.43 × 10^−3^	0.979
		PCP	0.0038 ± 0.001	−1.85 × 10^−3^ ± 1.33 × 10^−3^	0.958
		2,4-DCP	0.0029 ± 0.000	−1.15 × 10^−3^ ± 5.77 × 10^−4^	0.918
		4-CP	0.0046 ± 0.001	−1.50 × 10^−3^ ± 3.95 × 10^−4^	0.982
	0.85	Positive control	0.0040 ± 0.0007	−5.73× 10^−3^ ± 7.04 × 10^−4^	0.974
		PCP	0.0024 ± 0.0002	−1.74 × 10^−2^ ± 1.77 × 10^−2^	0.957
		2,4-DCP	0.0027 ± 0.0002	−2.90 × 10^−2^ ± 2.13 × 10^−2^	0.930
		4-CP	0.0029 ± 0.0001	−1.59 × 10^−3^ ± 1.02 × 10^−4^	0.951
	0.45	Positive control	0.0037 ± 0.0003	−6.54 × 10^−3^ ± 1.10 × 10^−3^	0.980
		PCP	0.0015 ± 0.0000	−0.0245 ± 0.0022	0.949
		2,4-DCP	0.0017 ± 0.0000	0.00732 ± 0.0055	0.857
		4-CP	0.0020 ± 0.0001	1.29 × 10^−3^ ± 2.68 × 10^−3^	0.909

^#^ *k*_1_ and *k*_2_ were the pseudo-first-order rate constants for the conversion of resazurin to resorufin and resorufin to dihydroresorufin, respectively. This was expressed as the mean ± standard deviation of three parallel data sets. * The mean coefficients were calculated by fitting three replicates to Equation (1) (n = 9, critical *R*^2^ = 0.636, *p* = 0.01 for *E. faecalis*; n = 13, critical *R*^2^ = 0.467, *p* = 0.01 for *E. coli*).

**Table 2:** The *k*_1_ and *k*_2_ values of the resazurin reduction model fitted by *E. faecalis* and *E. coli* to different heavy metals.

Bacteria	Group	*k*_1_ ^#^	*k*_2_ ^#^	*R*_2_ *
*E. faecalis* (n = 9)	Positive control	0.0366 ± 0.0023 ^a^	7.10 × 10^−21^ ± 2.34 × 10^−3^	0.977
	Pb	0.0084 ± 0.0004 ^b^	8.45 × 10^−3^ ± 3.97 × 10^−3^	0.991
	Zn	0.0076 ± 0.0001 ^c^	7.62 × 10^−3^ ± 1.48 × 10^−4^	0.983
	Cu	0.0074 ± 0.0001 ^c^	7.44 × 10^−3^ ± 1.02 × 10^−4^	0.982
*E. coli* (n = 13)	Positive control	0.00397 ± 0.0000 ^a^	4.07 × 10^−3^ ± 2.59 × 10^−5^	0.976
	Pb	0.00181 ± 0.0003 ^b^	−3.00 × 10^−2^ ± 1.17 × 10^−2^	0.914
	Zn	0.00199 ± 0.0001 ^b^	−4.52 × 10^−3^ ± 8.10 × 10^−4^	0.813
	Cu	0.00250 ± 0.0003 ^b^	7.35 × 10^−3^ ± 9.23 × 10^−3^	0.944

^#^ *k*_1_ and *k*_2_ are the pseudo-first-order rate constants for the conversion of resazurin to resorufin and resorufin to dihydroresorufin, respectively. This is expressed as the mean ± standard deviation of three parallel data sets. * The mean coefficients were calculated by fitting three replicates to Equation (1) (n = 9, critical *R*^2^ = 0.636, *p* = 0.01 for *E. faecalis*; n = 13, critical *R*^2^ = 0.467, *p* = 0.01 for *E. coli*). a, b, c: Significant difference labels. Turkey test in Anova (alpha = 0.05) was conducted on *k*_1_ of the experimental and control groups.

## Data Availability

The experimental data is contained within the article.

## Supplementary Materials

The following supporting information can be downloaded at: https://www.mdpi.com/article/10.3390/bios13050523/s1, Video S1: The PAD colorimetric response of *E. faecalis* to chlorophenol stress (pentachlorophenol, 2,4-dichlorophenol, and 4-chlorophenol) was sped up by a factor of 600 in the video. Video S2: The PAD colorimetric response of *E. coli* to chlorophenol stress (pentachlorophenol, 2,4-dichlorophenol, and 4-chlorophenol) was sped up by a factor of 600 in the video. Video S3: The PAD colorimetric response of *E. faecalis* to heavy metal stress (Cu^2+^, Zn^2+^, and Pb^2+^) was sped up by a factor of 600 in the video. Video S4: The PAD colorimetric response of *E. coli* to heavy metal stress (Cu^2+^, Zn^2+^, and Pb^2+^) was sped up by a factor of 600 in the video.
